# Novel Hybrid Treatment for Pulmonary Arterial Hypertension with or without Eisenmenger Syndrome: Double Lung Transplantation with Simultaneous Endovascular or Classic Surgical Closure of the Patent Ductus Arteriosus (PDA)

**DOI:** 10.3390/jcdd9120457

**Published:** 2022-12-14

**Authors:** Tomasz Stącel, Paweł Sybila, Agata Mędrala, Marek Ochman, Magdalena Latos, Fryderyk Zawadzki, Anna Pióro, Piotr Pasek, Piotr Przybyłowski, Tomasz Hrapkowicz, Ewa Mroczek, Agnieszka Kuczaj, Grzegorz Kopeć, Roland Fiszer, Szymon Pawlak, Anita Stanjek-Cichoracka, Maciej Urlik

**Affiliations:** 1 Silesian Centre for Heart Diseases in Zabrze, Department of Cardiac, Vascular and Endovascular Surgery, and Transplantology, Medical University of Silesia, 40-055 Katowice, Poland; 2 Silesian Centre for Heart Diseases in Zabrze, Department of Cardiac Anaesthesia and Intensive Care, Medical University of Silesia, 40-055 Katowice, Poland; 3 First Department of General Surgery, Collegium Medicum of Jagiellonian University, 30-688 Krakow, Poland; 4 Institute of Heart Diseases, University Clinical Hospital Mikulicz Radecki in Wroclaw, ul. Borowska 213, 50-558 Wroclaw, Poland; 5 Pulmonary Circulation Centre, Department of Cardiac and Vascular Diseases, Jagiellonian University Medical College, John Paul II Hospital, 31-202 Krakow, Poland; 6 Silesian Centre for Heart Diseases in Zabrze, Department of Congenital Heart Defects and Pediatric Cardiology, Medical University of Silesia, 40-055 Katowice, Poland; 7 Department of Biophysics, Faculty of Pharmaceutical Sciences in Sosnowiec, Medical University of Silesia in Katowice, Jedności 8, 41-200 Sosnowiec, Poland

**Keywords:** pulmonary arterial hypertension, patent ductus arteriosus, heart transplantation, lung transplantation, ECMO, conditioning and bridging to lung transplantation, postoperative conditioning, Eisenmenger syndrome, PDA closure

## Abstract

Patients with pulmonary arterial hypertension (PAH) become candidates for lung or lung and heart transplantation when the maximum specific therapy is no longer effective. The most difficult challenge is choosing one of the above options in the event of symptoms of right ventricular failure. Here, we present two female patients with PAH: (1) a 21-year-old patient with Eisenmenger syndrome, caused by a congenital defect—patent ductus arteriosus (PDA); and (2) a 39-year-old patient with idiopathic PAH and coexistent PDA. Their common denominator is PDA and the hybrid surgery performed: double lung transplantation with simultaneous PDA closure. The operation was performed after pharmacological bridging (conditioning) to transplantation that lasted for 33 and 70 days, respectively. In both cases, PDA closure effectiveness was 100%. Both patients survived the operation (100%); however, patient no. 1 died on the 2nd postoperative day due to multi-organ failure; while patient no. 2 was discharged home in full health. The authors did not find a similar description of the operation in the available literature and PubMed database. Hence, we propose this new treatment method for its effectiveness and applicability proven in our practice.

## 1. Introduction

Heart–lung transplantation (HLTx) should be considered in severe pulmonary hypertension and heart failure. An alternative method is double lung transplantation (DLTx), which is associated with the repair of cardiac or extracardiac defects [1,2,3]. However, this method is reserved for patients who have not developed irreversible, end-stage heart failure and those who have no irreversible, severe structural heart changes. One diagnostic method that enables an insight into structural changes, intracardiac shunts, and cardiac function is magnetic resonance imaging (MRI), which has gained importance recently [4,5].

## 2. Case Presentation

Patent ductus arteriosus (PDA) is an extracardiac heart shunt defect that may lead to serious, progressive, and often irreversible cardiovascular diseases if left untreated. It causes blood flow from the high-pressure system of the aorta (Ao) to the low-pressure pulmonary artery (PA) bed. This, in turn, increases pulmonary vascular resistance (PVR) over time and creates persistent pulmonary arterial hypertension (PAH) [1,2,3,6]. The increase in PVR and progressive PAH leads to the reversal of the shunt over time, which is defined as Eisenmenger syndrome (ES) [6]. It is manifested by hypoxemia and central cyanosis. In patients with ES, isolated PDA closure is contraindicated as it is a specific safety “valve” for progressive right ventricular failure [7].

We present here two patients with PAH: (1) one case of PAH due to a congenital defect (PDA) with developed ES., and (2) one case of idiopathic PAH who also had coexistent PDA, but right-to-left shunt did not occur. Both patients showed symptoms of right heart failure and had to undergo a period of pharmacological bridging to transplantation, called cardiac conditioning. Ultimately, both underwent hybrid DLTx with simultaneous PDA closure.

This alternative to HLTx allows the patient’s heart to be preserved, complications of heart–lung transplant to be avoided, and the donor’s heart to be used in another recipient; thus, the already limited pool of heart donors was not limited [8,9,10,11].

The condition for the application of hybrid surgery is the identification of the potential of the right (RV) and left (LV) ventricles regenerating based on additional tests, such as echocardiography and magnetic resonance of the heart, which should exclude severe structural defects and extensive cardiac fibrosis, especially within the LV [12,13,14,15].

In both patients, veno-arterial (VA)-extracorporeal membrane oxygenation (ECMO) was used during surgery and maintained according to the protocol of cardiac conditioning in the postoperative period. Conditioning the heart with ECMO usage, especially the LV, has been introduced in clinical practice in Europe on a large scale by leading transplant centers at Hannover and Vienna [16,17]. This theory assumes that in the postoperative period, the main problem may be left ventricular diastolic insufficiency, which worked under a reduced preload through PVR. The LV may be unprepared for full load immediately after the operation; therefore, the ECMO system is used in the VA configuration for periods of various length, depending on the progress of its conditioning. The authors especially recommended the method of connecting the VA-ECMO to the patient, which, after the operation, allows for quick awakening, extubation, and rehabilitation of the patient (detailed description of the implementation in the text below).

### 2.1. Case 1

The female patient was diagnosed with PDA during the first month of life. At 17 months, the patient underwent PDA ligation surgery; however, due to acute postoperative drug-resistant pulmonary hypertension accompanied by cardiovascular decompensation, the PDA was reoperated and the reversal of the arterial duct ligation was performed on the next postoperative day. The diagnosis of pulmonary hypertension was made at this point and for the following years it worsened, leading to ES.

The patient was under outpatient cardiology care, but pulmonary hypertension treatment was started when the patient was admitted to the pediatric cardiology department with supraventricular tachycardia. Cardiac catheterization was performed for the first time during this admission and pulmonary hypertension and ES were confirmed. Therefore, dual oral therapy (sildenafil and bosentan) was started at the age of 16. Subsequent drugs from the pool of disease-specific drugs were systematically introduced. At that time, drugs for pulmonary hypertension reimbursed in children did not include prostacyclins; therefore, triple combination therapy could be introduced only after the age of 18. Initially, the patient was treated with inhaled prostacyclin—iloprost (the patient did not consent to parenteral administration of prostacyclin) and after 3 months this was changed to subcutaneous prostacyclin (treprostinil).

On 13 January 2020, when the patient was 21 years old, she was presented to the transplant team for qualification to HLTx or DLTx with simultaneous PDA closure. Transthoracic echocardiogram (TTE), cardiac MRI, 6 min walk test, laboratory tests, and thorough physical examination were reviewed. Moreover, REVEAL 2.0 score was calculated.

The echocardiographic examinations performed during the qualification process (from referral on 13 January 2020 to final qualification on 11 September 2020) showed that the patient had: a progressive enlargement of the right heart chambers, pressure overload with impaired systolic function of the RV, estimated pulmonary pressure of 122 mmHg, and parameters of LV function showing deterioration over time (systolic dysfunction) (Table 1). Simpson’s method was used to assess EF, but it should be remembered that in patients with PAH and significant dominance of the right ventricle, which compresses the left ventricle, the diastolic parameters may be falsified, and thus translate into the underestimation of LV EF.

The results of the laboratory tests also showed deviations, especially in terms of N-terminal pro-B-type natriuretic peptide (NT-proBNP) and the spontaneously elevated international normalized ratio (INR) level (see Table 2, line, referral).

**Figure 1:**
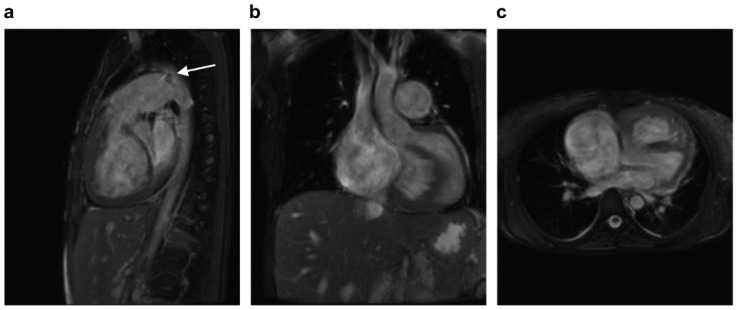
Presence of enlarged cavities of the right heart and liver. (**a**) Sagittal section, note the enlarged pulmonary artery with patent ductus arteriosus; the white arrow indicates patent ductus arteriosus; (**b**) front section, note the enlarged right cardiac chambers and liver; (**c**) transverse section, non-ischemic fibrosis of the left ventricle endocardium and spots of fibrosis within the right ventricle.

Cardiac MRI revealed the predominant pattern of LV fibrosis and circular LV endomyocardial fibrosis and less severe areas of fibrosis within the RV. Moreover, the right-to-left shunt through the PDA was documented, and LV and RV ejection fractions were reduced to 40% and 27% (normal value for RV is above 46% and for LV is above 55%), respectively (Figure 1a–c).

In the physical examination, peripheral cyanosis, ascites, and slight edema of the lower limbs were observed.

The REVEAL SCORE 2.0, which predicts the patient’s annual survival, was rated at 15 points, which means that the patient had a probability of survival within one year of <70% [18,19].

A total of 303 days after the qualification, the patient was admitted to the lung transplant department of our hospital with features of cardiovascular decompensation for pharmacological conditioning before DLTx: World Health Organization (WHO) class IV, ascites, and a significantly enlarged liver (documented in abdominal ultrasonography and MRI). Echocardiography revealed severe RV dilatation with impaired global systolic function and typical displacement of the interventricular septum (IVS) toward the LV (Table 1; column “at admission”). In laboratory tests, the concentration of NT-proBNP and level of INR were also elevated in comparison to the referral time (Table 2; line, admission to lung transplant centre).

In preparation for surgery, the patient underwent treatment aimed at reducing heart failure—catecholamine was introduced; dopamine (400 mg/50 mL–2 mL/h; 5.33 µg/kg/min), a loop diuretic, torasemide (200 mg/50 mL–1 mL/h; 1.33 µg/kg/min), and PAH-specific therapy were continued; phosphodiesterase-5 inhibitor (sildenafil) 20 mg three times a day, endothelin-1 receptor blocker (bosentan) 125 mg twice a day, and treprostinil subcutaneously at a dose of 136 ng/kg/min were administered (10 mg/mL; infusion rate of 42 µL/h).

Due to the patient’s cachexia, the caloric supply was increased, and an additional source of proteins was supplemented. The patient was examined by a psychologist as part of a crisis intervention due to low mood and depressive states. The physiotherapeutic team interviewed and implemented bedside rehabilitation.

**Figure 2:**
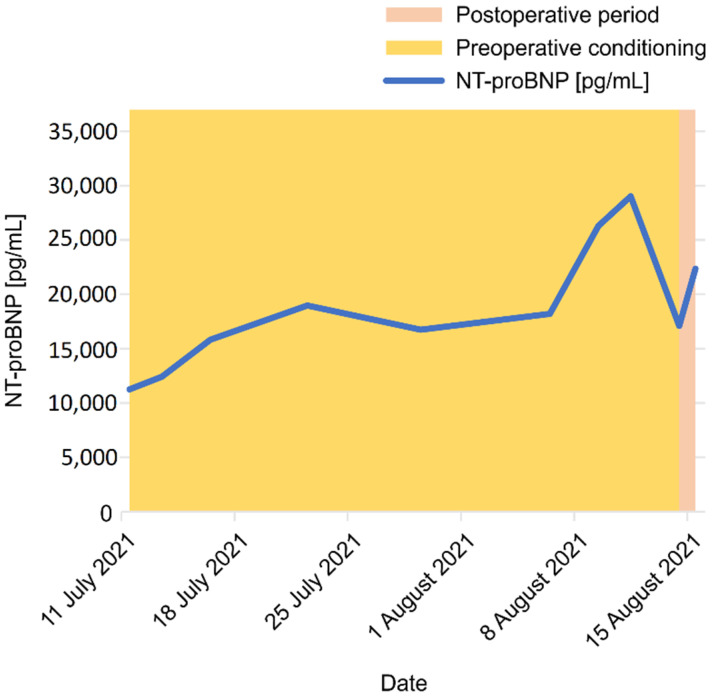
The concentration of NT-proBNP during the pharmacological conditioning period before lung transplantation, patient no 1 NT-proBNP, N-terminal pro-B-type natriuretic peptide.

The patient’s condition stabilized, and decompensation was categorized as WHO class III. The TTE performed after the conditioning period (immediately before the surgery) showed an improvement in the function of the LV and RV (measured by tricuspid annular plane systolic excursion), as well as the normalization of the fluid in the pericardium (Table 1; column “before surgery”). At the same time (during the conditioning period), no significant improvement was observed in the concentration of NT-proBNP (Figure 2).

On 13 August 2021, after 33 days of preoperative pharmacologic conditioning with a fairly good effect, the patient underwent surgery after finding a compatible donor.

The HLTx option was rejected due to the very high risk of intra- and post-operative bleeding and associated risk of death [20]. This risk included the following factors: liver failure, as evidenced by the spontaneously elevated INR (1.42); the need for systemic heparinization during extracorporeal circulation (cardiopulmonary bypass); multiple adhesions after previous surgery; and almost impossible revision when needed for potential bleeding in this area. At the same time, after a positive assessment of the chances of heart regeneration (based on MRI and TTE examinations), especially if LV conditioning techniques were taken into account (prolonged use of ECMO-VA in the postoperative period), the patient was qualified for hybrid DLTx with simultaneous endovascular PDA closure.

**Figure 3:**
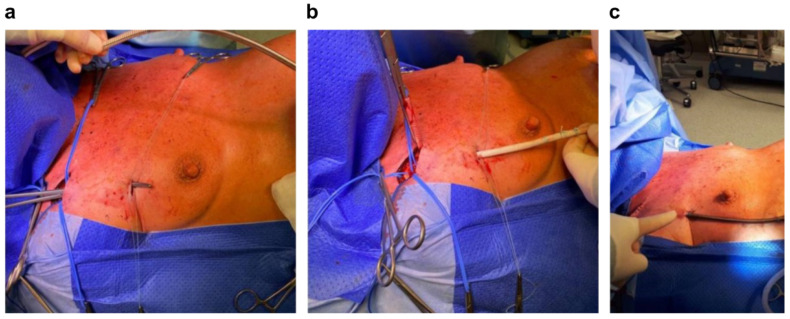
(**a**) Incision in the right subclavian artery creating a tunnel under the pectoral muscle; (**b**) passing a vascular prosthesis through the tunnel and suturing it to the subclavian artery; (**c**) inserting an extracorporeal membrane oxygenation delivery (“arterial”) cannula into the vascular prosthesis.

Due to significant pulmonary hypertension (suprasystemic) in the course of ES, the procedure was performed with VA-ECMO support. We used upper body ECMO, which is sometimes called “Sport ECMO” because it enables quick activation, upright standing, and rehabilitation in the postoperative period as it does not require femoral access (Figure 3) [21,22]. For this purpose, a parallel incision to the right subclavian was performed (Figure 3a), the subclavian artery was dissected, and an 8Fr vascular prosthesis was implanted (Figure 3b) to which the inflow cannula of the ECMO system was connected (Figure 3c). The receiving cannula of the ECMO system was introduced into the right atrium (RA) by the Seldinger technique through the right internal jugular vein. It should be remembered that lung resection can only be started after ECMO is launched. VA-ECMO prevents an increase in resistance to the weakened RV after clamping one of the pulmonary arteries, which is performed just before lung resection.

**Figure 4:**
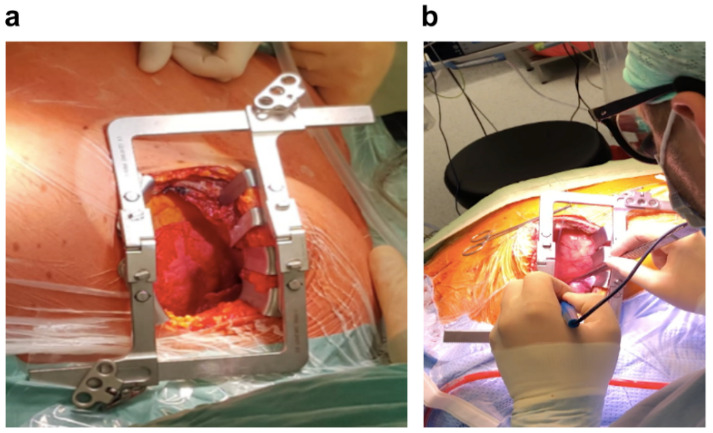
(**a**,**b**) Anterolateral mini-thoracotomy.

The procedure itself was started with left-sided mini-thoracotomy (Figure 4) and numerous adhesions (after two interventions in childhood—see above) were released—preparation causing profuse bleeding (cell saver was used). After reaching and dissecting the hilum, ECMO was started and the left pulmonary artery was clamped, pulmonary veins were ligated, and finally the left lung was resected.

**Figure 5:**
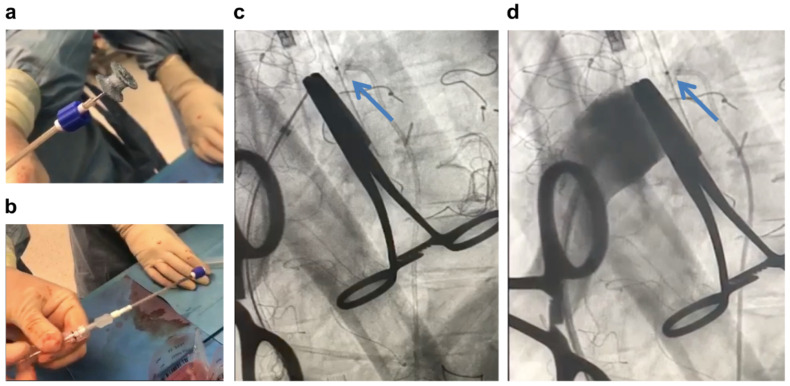
(**a**) AMO, Amplatzer™ Muscular Occluder before being rolled up to the vascular sheath; (**b**) AMO in delivery system—vascular sheath ready for insertion; (**c**) implanted and opened AMO; (**d**) angiography, no leakage; the blue arrows indicate the aortic portion of the AMO.

After complete hemostasis was achieved, the endovascular stage was started: the femoral artery and vein were punctured. The catheter, wire, and subsequently the sheath were inserted into the RV and PA and through PDA into the Ao. A 6 mm Amplatzer™ Muscular Occluder (AMO) was implanted through venous access. Check-up angiography was performed from the Ao and PA, which showed complete PDA closure with no residual leak *(*Figure 5).

After the endovascular part was completed, bronchial anastomosis was performed first, then arterial and venous anastomosis, vascular clamps were removed, and reperfusion and ventilation of the transplanted lung were initiated.

On the right side, the essential elements of the operation were the same. Access was obtained via mini-thoracotomy, but the preparation of the right lung was much easier due to the lack of adhesions in the pleural cavity, while due to the features of a hemorrhagic diathesis after the operation on the left side, the patient required a constant supply of fluids, including blood products, in order to maintain the correct ECMO output. After bronchial anastomosis, an anastomosis of the PA and then of the pulmonary veins was performed, and perfusion and ventilation of both lungs were started. The drains were placed and the chest closed.

After 22 h of surgery with a properly functioning pulmonary graft, the patient was transferred to the intensive care unit in an intermediate condition. This condition resulted from the length of the surgical part of the operation, and especially from difficulties in the preparation of adhesions accompanied by bleeding. It should be remembered that as part of specific PAH therapy, the patient was receiving treprostinil, which may impair platelet count and function [23,24,25]. On the other hand, it should be emphasized that the endovascular part of the operation went as planned without any complications, was absolutely effective (due to the fact that the operation was performed in a hybrid room, it was demonstrated using intraoperative angiography), and lasted only 30 min.

In the postoperative period, with normal graft function, VA-ECMO was maintained in accordance with the cardiac conditioning protocol, but the patient required high doses of vasopressin 0.001 IU/kg/min, adrenaline (from 0.13 µg/kg/min up to 0.325 µg/kg/min), and noradrenaline 1.3 µg/kg/min according to arterial pressure. Despite the treatment, multiple organ failure (MOF) occurred and worsened quickly. The probable cause of MOF in the postoperative period was systemic inflammatory response syndrome (SIRS) related to the extent and duration of the operation; increased intra- and postoperative bleeding; blood circulation in the ECMO artificial circuit, which causes the activation of a number of cytokines; and ultimately could lead to septic shock, taking into account the immunosuppressive treatment used. On 15 August 2021, after 2 days of surgery, the patient died of a lytic drop in blood pressure that did not respond to the administered drugs.

**Figure 6:**
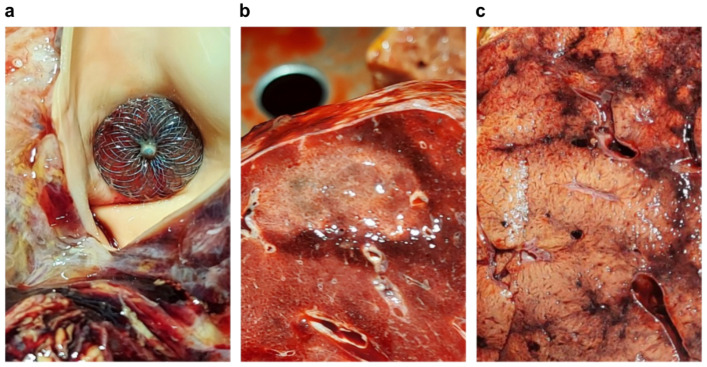
Postmortem pictures from autopsy; (**a**) Correct position of the implanted Amplatzer™ Muscular Occluder; (**b**) liver parenchyma with dilated hepatic veins and focal necrosis; (**c**) pale, ischemic, and necrotic hepatic lobule.

The autopsy revealed central and lobule necrosis of the liver (Figure 6), fibrinous epicarditis, and features of hemorrhagic diathesis in the form of extravasation in the subcutaneous tissue, as well as, importantly, the correct position of the implanted AMO.

### 2.2. Case 2

The case presents a 39-year-old female patient with PAH and coexistent PDA, which, due to its size, could not be the direct cause of PAH. As the disease progressed and PVR increased, a slight shunt through PDA from the right to the left side of the circulation developed as a kind of safety “valve” for the deteriorating RV function.

At the time of diagnosis of the disease in April 2019, pharmacotherapy with bosentan was started, and after 5 months (in September 2019), the patient was already receiving triple therapy (bosentan, sildenafil, and treprostinil). Due to the progressing disease, bosentan was changed to macitentan in the later stages of the treatment.

The patient was admitted to the lung transplant department on 31 August 2021, with symptoms of severe decompensated RV failure, i.e., ascites, edema of the lower limbs, and clinical symptoms of WHO class IV progressing to dyspnea at rest.

**Figure 7:**
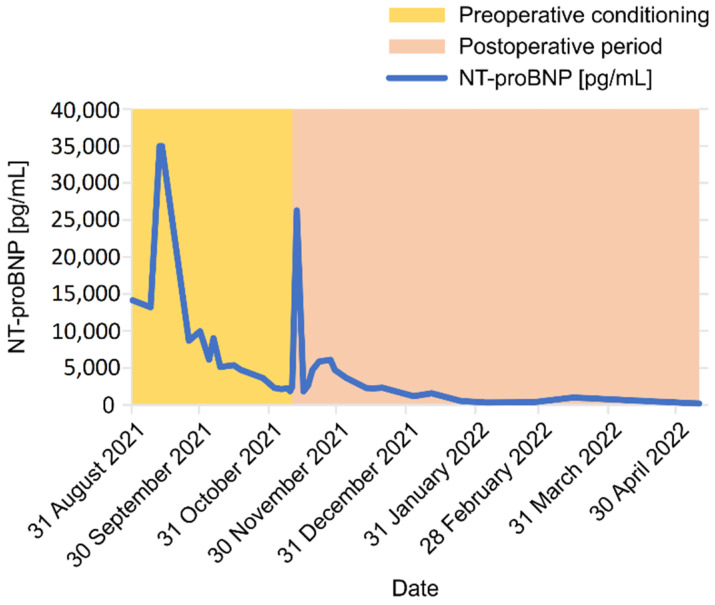
The change in NT-proBNP concentration in the period from admission to postoperative follow-up for 6 months NT-proBNP, N-terminal pro-B-type natriuretic peptide.

Echocardiographic examination revealed features of PAH, the most important of which were the RV dilatation with severe secondary tricuspid valve insufficiency and permanent displacement of the IVS toward the LV. The results of the TTE test at admission and at the later stages of treatment are summarized in Table 3. Moreover, biochemical parameters, such as increased NT-proBNP concentration reaching 5311 pg/mL and creatinine concentration of 168 µmol/L (estimated glomerular filtration rate of 31 mL/min/1.73 m^2^), as well as spontaneously elevated INR to 1.39, indicate a decompensation of the patient’s condition (Table 4 and Figure 7).

The patient’s REVEAL SCORE 2.0 in this condition amounted to 16 points, meaning that the probability of survival within one year was very low and less than 70%.

The above decompensation occurred despite the use of diuretic treatment (furosemide 100 mg/50 mL) and the maximum doses of specific therapy: sildenafil 3 × 20 mg daily orally, macitentan 10 mg daily orally, and treprostinil 63 ng/kg/min into the central vein using a Hickman catheter and a personal infusion pump.

**Figure 8:**
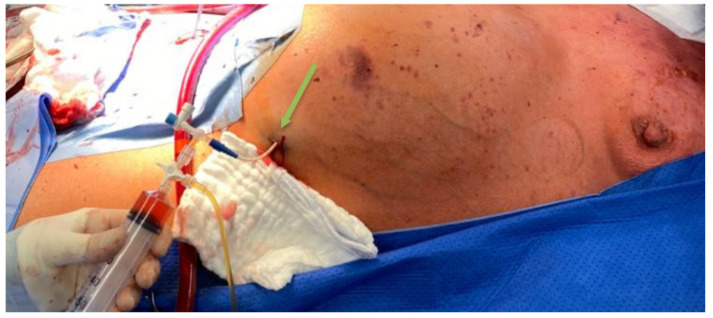
Drainage of ascites fluid from the abdominal cavity; green arrow indicates vascular catheter.

Despite the introduction of pressor amines (dobutamine 13.8 µg/kg/min and noradrenaline from 0.014 µg/kg/min up to 0.083 µg/kg/min, the dose of which depended on blood pressure) and intensified diuretic treatment, cardiovascular decompensation progressed. Furthermore, renal failure required renal replacement therapy in the form of continuous veno-venous hemodialysis. In addition, there was an episode of infection with symptoms of sepsis with C-reactive protein (143.4 mg/L), procalcitonin (2.6 ng/mL), and significant hypotension (arterial pressure: 50/30 mmHg) persisting for several days. Due to the suspicion that the focus of sepsis may be pneumonia, the patient was initially treated empirically and then with targeted antibiotic therapy (meropenem 500 mg IV, two times a day and cloxacillin 1 g, three times a day). The regular daily drainage of ascites fluid from the abdominal cavity (Figure 8) was started using a vascular catheter with simultaneous intravenous albumin infusion to maintain normal oncotic pressure inside the vessels to reduce the risk of ascites recurrence.

At this stage, the patient was not qualified for LTx nor HLTx due to the high risk of complications and death in the perioperative period. Moreover, if there was no improvement in the above treatment, the management of mechanical circulatory support in the form of VA-ECMO was considered as a bridge (conditioning) to transplantation.

Due to cachexia and severe general conditions with accompanying anemia, the patient required an increased supply of caloric and qualitative meals, transfusion of blood products, and erythropoietin, which had a very good effect. A continuous infusion of albumin was maintained to increase protein concentration and optimally fill the vascular bed.

After a few days of applying the above changes, the conversion of dobutamine to dopamine 5.33 µg/min/kg, introduction of antibiotic therapy, and bedside rehabilitation, the patient’s general and hemodynamic condition improved. Along with the treatment, the laboratory parameters also improved (Table 4): all of NT-proBNP, INR, and cystatin were significantly reduced (Table 4 and Figure 7). Regularly performed cultures of biological samples (blood, urine, and sputum) were negative. In this condition, the patient underwent a positive re-evaluation in terms of qualification for LTx or HLTx.

Due to the predicted chance for regeneration of the RV and LV based on the improvement of echocardiographic parameters (tricuspid annular plane systolic excursion, resolution of pericardial effusion showing a trend towards improvement) in the conditioning period (compared to echocardiography on admission) (Table 3), the option for DLTx with simultaneous closure of the PDA was selected in this patient. In particular, the chance of myocardial regeneration was expected with the use of a cardiac conditioning protocol with prolonged VA-ECMO in the postoperative period [16,17,26].

On 9 November 2021, after 70 days of preoperative conditioning (bridging), DLTx with the use of VA-ECMO was performed along with the classical surgical closure of the PDA.

As already mentioned, VA-ECMO implantation is the first stage of LTx in patients with PAH. The authors’ center uses a variety of upper-body ECMO due to the possibility of quick awakening, extubation, and mobilization of the patient, including full rehabilitation. The method of implantation of an arterial inflow cannula was performed in a similar manner to that described above (case No. 1). The VA-ECMO output was started at 3 L/min according to the LV conditioning protocol and continued in the postoperative period.

After ECMO implantation, the proper stage of the lung transplant operation was performed via mini-thoracotomy. The left lung hilum structures were dissected, the pulmonary artery was clamped, the pulmonary veins were ligated, and then the left lung was excised. At this stage, the PDA was surgically dissected, ligated, and clipped.

First, bronchial anastomosis was performed, followed by arterial and venous anastomosis, then the vascular clamps were removed, and reperfusion and ventilation of the transplanted lung were started. On the right side, the operation was performed in the same way. The drains were placed and the chest closed. The LV conditioning protocol with VA-ECMO followed, and the patient was transferred to the intensive care unit in a stable condition.

During regular echocardiography (from 1 to 2 times a day), depending on the test result and hemodynamic status, it was possible to systematically reduce the ECMO output from 0.5 L/min/day to 0.7 L/min/day, and thus condition the LV. It also showed a systematic improvement in right ventricular function.

The passive and then active rehabilitation process was begun. The patient was weaned from ECMO and extubated on days 8 and 10 after surgery, respectively. The kidneys improved with the return of efficient diuresis within one month after transplantation (Table 4).

Despite typical immunosuppressive treatment after transplantation (tacrolimus 0.01 µg/min/kg, mycophenolate mofetil 2 × 500 mg, and prednisolone 20 mg once daily) and the use of preoperative induction immunosuppression with basiliximab, acute rejection occurred on the 8th postoperative day. To counteract it, the patient was immediately given an intravenous infusion of polyclonal antibodies (intravenous immunoglobulin) and three treatments of therapeutic plasmapheresis. The patient was also treated with pulses of methylprednisolone of 30 mg once a day with good results.

Postoperative bronchoscopic controls showed proper healing of the anastomoses. As with most of the transplanted patients, she also required transfusions of blood products several times in the postoperative period.

In good general condition, the patient was discharged home 45 days after the procedure.

To the day of submission of this manuscript, the patient is under the constant supervision of our transplant center. In echocardiographic examinations performed at the 3rd and 9th months after the procedure: the heart dimensions are normal, and there is a good global systolic–diastolic function of the LV and RV. The heart valve apparatus is normal without regurgitation and structural changes. No pathological fluid in the pericardium is noticed (Table 3). In laboratory tests, the level of NT-proBNP and other biochemical parameters decreased and remain within normal values (Table 4 and Figure 7).

## 3. Discussion

The article describes a new surgical technique in the treatment of PAH with associated PDA. It presents the development and course of the disease from birth to adulthood, the pharmacological and surgical treatment attempts undertaken at that time, and the process of qualification for LTx with its culmination in the form of a new surgical technique.

The descriptions of surgical procedures used in elderly patients with PAH and congenital defect with and without ES are rare.

PAH is a rare disease and is characterized under orphan diseases. Depending on the country and region, and due to the heterogeneity, the prevalence of PAH is reported to be between 2 to 15 per million inhabitants [27].

The first group of pulmonary hypertension, according to the classification proposed by WHO, is very diverse (Table 5) and includes both patients with iPAH, as well as cases in which PAH develops due to congenital defects, connective tissue diseases, drugs, or genetic factors. The subgroup with existing shunt defects and/or ES is even smaller.

After analyzing the databases, it should be concluded that, first of all, there are very few descriptions of LTx operations with simultaneous correction of a shunt defect (atrial septal defect (ASD), ventricular septal defect (VSD), and PDA) or HLTx. Secondly, the comparative results of both methods (DLTx vs. HLTx) were often contradictory for many years [1,2,3,28,29]. Our descriptions of operations in patients with PAH and PDA (additionally with developed ES, such as in case no. 1) fill this gap in the current medical literature. To the best of our knowledge, this is a pioneering study. In particular, the method with endovascular closure of PDA is an innovative approach to the subject using advanced techniques and medical devices concentrated within a hybrid room.

In the intention of the authors, the second of the presented cases was also to be carried out with the use of endovascular techniques in the hybrid room, but the lack of availability of the hybrid room on that day forced the authors to use a classic ligation procedure and cut the PDA.

LTx or simultaneous HLTx remain the last option in patients who are no longer responding to treatment, most often in WHO class III or IV, with heart failure and secondary failure of other organs.

Regardless of statistical data, it has been believed for many years that patients who have a weak RV with no prognosis of regeneration are not suitable for LTx and, therefore, should be referred for a simultaneous HLTx.

However, the results of various studies, as well as regular International Society for Heart and Lung Transplantation (ISHLT) reports, indicate that survival, if not inferior, is even better after LTx, compared to simultaneous HLTx for PAH. A study by Kearney [30] compared these two transplant strategies in ES. It turned out that in recent years (2005–2018), patients with ES in the course of ASD who underwent LTx surgery with heart repair had a better 1-, 5-, and 10-year survival than patients after HLTx. Additionally, the advantage of DLTx with heart repair, compared to HLTx, is the possibility of using a donor’s heart in a different recipient. Therefore, as recommended since 2010, DLTx over HLTx is the recommended surgical treatment for PAH [31]. Additionally, the waiting time for a donor is longer with HLTx [32]. According to the same sources, the number of simultaneous heart and lung transplants (HLTx) dropped over the period of 1990–2000, of which only one-third concerned the idiopathic variant of PAH. This is also the case in the studies that suggest there is significant regenerative potential of the RV after LTx alone [32,33,34,35].

The results of DLTx are significantly better compared to single lung transplantation (SLTx) in terms of both early survival (because the risk of primary graft dysfunction is reduced) and long-term survival [36]. For this reason, SLTx should be restricted to genuinely exceptional cases or banned altogether in this disease entity.

In recent years, several drugs, both oral and intravenous, known collectively as specific therapy, have entered the market, significantly improving patients’ hemodynamic status and prolonging survival. New therapies, such as sotatercept, are already waiting in line to be included in the pharmacological armamentarium used in the treatment of PAH [37,38].

In the event of an inadequate response to pharmacological treatment, invasive therapies, such as Potts anastomosis, historical balloon atrial septostomy, and newer ones, such as atrial flow regulators (AFR), remain in practice [39,40,41]. The results of AFR research are awaited, which could shed new light on current treatments [42].

For various reasons, including the initially good pharmacological response, patients with PAH are referred to transplant centers too late when, secondary to the RV failure, there is damage to other organs, such as the liver and kidneys. There is a group of patients that go to transplant centers with ascites, edema, onset of cachexia, and secondary thrombocytopenia. As shown in several studies, thrombocytopenia may have a significant impact on perioperative and long-term survival [23,24,25].

Moreover, the process of qualifying patients within transplant departments may be extended, and the decision to qualify may also be difficult when the patient presents the above symptoms of deterioration, each of which falls within the category of relative or absolute contraindications for transplantation.

Additionally, in the cases presented, it seems that the referral and qualification process, especially in case no. 1, should be shortened, which is in line with the idea suggested by Quezada–Loaiza et al. [28]. The patients referred to our center had a high result in the REVEAL score 2.0 scale, which meant that they presented a very low probability of one-year survival—less than 70% [43].

However, the qualification process carried out above, culminating in LTx, should be appreciated because, as de Perrot [29] states, only about 30% of PAH-referred patients are eventually lung transplanted.

Each exacerbation of the disease, resulting, for example, from an infection of the respiratory system or deterioration of the functions of other organs, rarely causes these patients to return to their previous functional state, and this irretrievably causes the shrinking of the already narrow transplant window. Both of the presented patients experienced such exacerbations several times in the course of their disease, which ultimately led to the need for hospitalization in our ward for organ conditioning before performing a hybrid transplantation of both lungs with simultaneous closure of the patent PDA.

Despite the introduction of organ allocation systems in Europe and the United States, PAH recipients are still not considered first-choice candidates; hence, their long stay on the waiting list, systematic deterioration of their health, and death while waiting [8,9,10,11]. In one study, 8 of 20 patients who qualified for transplantation had lung and heart transplants (HLTx), but the mortality rate was high, and more than half of them died within one week to four years. Five patients died while waiting; four were disqualified for various reasons, including serious deterioration of their health. Three patients were still on the waiting list but are unlikely to undergo transplantation due to systematically deteriorating health—mainly deteriorating kidney function [44].

In the authors’ opinion, the above-mentioned invasive therapies (balloon atrial septostomy, AFR, pulmonary artery denervation, Potts shunt) and, additionally, mechanical techniques of circulatory support, at the top of which is ECMO, may be of additional importance for such patients. These serve as bridging therapies for LTx [16,17,26,27,41].

The period of stay of these patients in our transplantation department was used for the so-called conditioning at the organ and systemic level. Conditioning included: improvement of heart function with catecholamines (mainly dopamine or dobutamine, but also adrenaline or noradrenaline); improvement of blood pressure (with vasopressin), especially in the septic period as in the case no. 2; fighting infections with targeted antibiotic therapy; and improvement of kidney function with the use of renal replacement therapy. It should be remembered that elevated creatinine levels are an independent risk factor for death in patients with ES [44]. Iron and platelet count and function were regularly checked and replenished as its deficiency may have an adverse impact on intraoperative and postoperative risk of bleeding [23,25,45,46,47]. It is worth noting that the specific therapy itself has depleting potential with regard to the number and functions of platelets [23,24,25]. Each time before any surgery, this function should be checked. In the presented patients, due to the improvement of their general condition and the improvement of biochemical parameters, there was no need to use the preoperative ECMO system.

The assessment of the reversibility of heart changes is a key factor in determining the feasibility of LTx with heart repair. The standard examination in this aspect is echocardiography. According to the latest research, magnetic resonance imaging (MRI) is gaining recognition as a prognostic tool for assessing the regenerative capacity of the heart muscle. In addition to purely diagnostic information, MRI also brings a prognostic element: an increasing number of authors pay attention to the relationship between the function of the ventricles and the degree of fibrosis of the RV and LV [48]. The degree of severity of myocardial fibrosis may indicate non-regenerative capacity of the heart chambers in the postoperative period, and thus may influence decisions regarding the choice of treatment method or the possibility of surgical treatment in general [5,48,49,50].

It should be remembered that the operation performed in a person with long-term ES, compared to surgery in a patient with iPAH, carries a higher risk of adverse events, mainly due to the fact that patients with ES suffer from birth, and, therefore, their internal organs have been damaged since childhood. There are opinions to treat ES as a multi-system disorder due to the fact that it affects the functioning of many organs [51]. Most of the authors dealing with this subject point out that the results of LTx or HLTx are unsatisfactory, mainly due to delayed qualification, long wait lists for an organ, and the resulting organ complications—liver and kidney, which translate into operational results [44]. In the study of Haworth and Hislop [52], it is noted that patients with corrected heart defect and recurrent or persistent pulmonary hypertension do worse than patients with ES or even with iPAH [51]. It should be mentioned that in patient No. 1 during childhood, an attempt was made to correct the PDA, but due to hemodynamic decompensation caused by pulmonary hypertension, a reversal of the arterial duct ligation was performed. This operation performed in childhood should be considered as an additional risk factor. Lung transplants in patients after previous surgical interventions in the area of the thorax, pleura, or lungs are the most demanding from a technical point of view and, as numerous analyses have shown, they represent a statistically significantly increased risk of complications (bleeding, nerve injury, and internal organ injury—liver and renal insufficiency), including death [20]. This explains why we chose endovascular access in the first described patient. This is also probably the reason why reoperations and retransplants are one of the least frequently performed transplant operations in the world, not exceeding 4% in ISHLT reports. In the second patient, due to the lack of previous cardiac surgery and no contraindications and the lack of availability of the hybrid room on the operation day, the surgical PDA ligation procedure was performed.

In the literature, one can find a division in ES with a simple heart defect (ASD, VSD, PDA) and a complex heart defect, such as complete transposition of large arteries and ventricular septal defect, complete atrioventricular canal defect, double RV outlet, single-chamber hearts, common trunk, and the like [44].

Therefore, the cases presented by us should be classified into the first group—“simple heart defect”. According to the data from the work by Daliento et al., patients with ES due to complex heart defects experience earlier clinical deterioration than those with simple defects (mean age 18.6 ± 11.3 vs. 26.7 ± 12.2 years, *p* < 0.001) [44]. Both of our presented patients with simple cardiovascular defects were in the age of 22 and 39 years; hence, the long-term influence of the defect led to such advanced changes in the functioning of organs (liver and kidneys) and in their functional state.

Although case No. 1 shows the possibilities of the modern treatment of ES with a hybrid method, i.e., simultaneous transplantation of both lungs using the latest endovascular techniques for PDA closure, case No. 2 shows the process of exemplary preoperative pharmacological conditioning of a patient for LTx, also performed with a hybrid technique; however, in this case, performed with the aid of the classic PDA closure.

It is, therefore, worth having the ability of the above-described hybrid treatment in armamentarium, remembering that it is possible after meeting certain criteria, the most important of which is the chance for heart regeneration in the postoperative period, which should be confirmed, among others, by MRI or echocardiography.

## 4. Conclusions

Patients with PAH constitute the most difficult group of patients, besides retransplantation, referred to transplant centers. Although PAH belongs to the so-called orphan diseases with a frequency of 30 cases per million adult population in the authors’ country, it is a heterogeneous entity, which makes the diagnostic and therapeutic process difficult [53]. Patients with accompanying heart defects, such as PDA, may develop PAH. Moreover, ES (patient no. 1) or a mild heart defect (PDA) may be accidentally discovered during the diagnosis of developed iPAH (patient no. 2). Although specific therapy relieves symptoms and extends the life of patients, a large group of these patients eventually stop responding to increasing doses of drugs and require transplantation treatment. Based on the results of scientific works and data from registries, such as the ISHLT, the RV has a high regenerative capacity after LTx, which means that the currently preferred method of surgical treatment is DLTx and not HLTx. The solution presented here, including simultaneous transplantation of both lungs with heart defect correction (PDA), especially with the endovascular variant, is an innovative approach.

The authors of the article did not find a similar description of the operation in the available literature and after analyzing the PubMed database. Hence, we propose treating patients with PAH and PDA with the above-mentioned new treatment method, as its effectiveness and applicability have been proven in our practice. Moreover, it supplements the gap in the literature. The article serves as a concise summary of treatment options for patients with PAH and ES in relation to PDA. It indicates critical diagnostic moments and clinical decisions made, as well as showing both the advantages and disadvantages of the procedures performed.

## Figures and Tables

**Table 1:** Echocardiographic parameters during the qualification process (from referral to final qualification), conditioning period before operation, and 1st postoperative day.

Patient No. 1	Referral and Qualification for LTx	Preoperative Conditioning, after Qualification	
		At admission	Before surgery
LV EF (%)	66	45	53
IVC insp. (mm)	18	25	LoD
IVC exp. (mm)	18	20	LoD
RVSP (mmHg)	107	125	126
RVFAC (%)	17	13	LoD
Ring TV (mm)	LoD	55	50
TAPSE (mm)	14	8	11
Pericardial effusion	NO	YES	NO
Leftward ventricular septum shift	YES	YES	YES
Severe tricuspid valve regurgitation	NO	YES	YES

Abbreviations: LoD, lack of data; LV EF, left ventricular ejection fraction; IVC, inferior vena cava; RA, right atrium; RVSP, right ventricular systolic pressure; TV, tricuspid valve; TAPSE, tricuspid annular plane systolic excursion; LTx, lung transplantation; insp., inspiratory; exp., expiratory; RVFAC, right ventricular fractional area change.

**Table 2:** Laboratory tests.

Laboratory Tests						
Date	NT-proBNP (pg/mL)	Albumin (g/L)	Creatinine (µmol/L)	Cystatin C (mg/L)	eGFR (mL/min/1.73 m^2^)	INR
Referral	4353	35	81	LoD	>60	1.24
Admission to LTC (for conditioning—bridging to LTx)	11,251	39	60	1.08	>60	1.42
Before surgery	29,028	51	113	1.88	56	1.34
After surgery	22,372	29	135^1^	LoD	45 ^1^	2.86

Abbreviations: LTC, lung transplant centre; LTx, lung transplantation; LoD, lack of data; NT-proBNP, N-terminal pro-B-type natriuretic peptide; CVVHD, continuous veno-venous hemodialysis; eGFR, estimated glomerular filtration rate; INR, international normalized ratio. ^1^ on CVVHD.

**Table 3:** Laboratory tests. Echocardiographic parameters from qualification through conditioning to postoperative follow-up (6 months).

Patient 2	Qualification 31 August 2021	Before Surgery—Preoperative Conditioning	1 Month after Surgery	3 Months after Surgery	6 Months after Surgery
LV EF (%)	44	42	55	55	52
IVC insp. (mm)	23	22	LoD	14	7
IVC exp. (mm)	29	27	LoD	23	14
RVSP (mmHg)	105	115	LoD	36	LoD
RVFAC (%)	16	19	LoD	35	30
Ring TV (mm)	45	53	LoD	LoD	LoD
TAPSE (mm)	8	14	25	22	25
Pericardial effusion (YES/NO)	YES	NO	trace separation of pericardial layers in systole	NO	NO
Leftward ventricular septum shift (YES/NO)	YES	YES	NO	NO	NO
Severe tricuspid valve regurgitation (YES/NO)	YES	YES	NO	NO	NO

Abbreviations: LoD, lack of data; LV EF, left ventricular ejection fraction; IVC, inferior vena cava; RA, right atrium; RVSP, right ventricular systolic pressure; TV, tricuspid valve; TAPSE, tricuspid annular plane systolic excursion; LTx, lung transplantation; insp., inspiratory; exp., expiratory; RVFAC, right ventricular fractional area change.

**Table 4:** Laboratory tests; from qualification through conditioning to postoperative follow-up (6 months).

Laboratory Tests						
Date	NT-proBNP (pg/mL)	Albumin (g/L)	Creatinine (µmol/L)	Cystatin C (mg/L)	eGFR (mL/min/1.73 m^2^)	INR
Referral, admission to LTC (31 August 2021)	14,135	45	168	LoD	31	1.39
Before surgery	1859	52	64 ^1^	1.85	>60 ^1^	1.16
After surgery (1st day)	2450	47	95	1.77	>60	1.2
After surgery (1st week)	26,278	38	106	1.69	53	1.12
After surgery (1st month)	2269	43	44	LoD	>60	1.13
After surgery (6th month)	184	39	59	1.26	>60	1.06

Abbreviations: LTC, lung transplant centre; LTx, lung transplantation; LoD, lack of data; NT-proBNP, N-terminal pro-B-type natriuretic peptide; CVVHD, continuous veno-venous hemodialysis; eGFR, estimated glomerular filtration rate; INR, international normalized ratio. ^1^ on CVVHD.

**Table 5:** Classification of PAH.

Pulmonary Arterial Hypertension
1.1 Idiopathic PAH
1.2 Heritable PAH
1.3 Drug- and toxin-induced
1.4 PAH associated with:
Connective tissue disease
HIV infection
Portal hypertension
Congenital heart diseases
Schistosomiasis
1.5 PAH long-term responders to CCBs
1.6 PAH with overt features of PVOD/PCH
1.7 Persistent PH of the newborn

Abbreviations: CCB, calcium channel blocker; PAH, pulmonary arterial hypertension; PCH, pulmonary capillary hemangiomatosis; PH, pulmonary hypertension; PVOD, pulmonary veno-occlusive disease; HIV, human immunodeficiency virus.

## Data Availability

Not applicable.
